# Association between housing status and mental health and substance use severity among individuals with opioid use disorder and co-occurring depression and/or PTSD

**DOI:** 10.1186/s12875-025-02947-2

**Published:** 2025-08-08

**Authors:** Lauren Kelly, Grace M. Hindmarch, Katherine E. Watkins, Colleen M. McCullough, Beth Ann Griffin, Lisa S. Meredith, Sapna Mendon-Plasek, Miriam Komaromy, Sarah B. Hunter

**Affiliations:** 1 RAND, Boston, MA USA; 2 RAND, Santa Monica, CA USA; 3 Department of Health Policy and Management, University of California, Los Angeles, Los Angeles, CA USA; 4 RAND, Arlington, VA USA; 5 Grayken Center for Addiction, Boston Medical Center, Boston, MA USA; 6 Division of General Internal Medicine, Chobanian and Avedisian School of Medicine, Boston University, Boston, MA USA

**Keywords:** Homelessness, Unstable housing, Housing stability, Opioid use disorder, Substance use disorder, Mental health, Post-traumatic stress disorder, Depression, Collaborative care

## Abstract

**Background:**

Opioid use disorder, mental health conditions, and housing instability are frequently intertwined and have a profound impact on health outcomes. While past research has focused on the opioid use and mental health of people experiencing homelessness, less is known about those experiencing housing instability. We examined the cross-sectional associations between housing status (currently unhoused, unstably housed, and stably housed) and mental health and substance use severity among primary care patients with co-occurring disorders.

**Methods:**

Data are from a randomized controlled trial, Collaboration Leading to Addiction Treatment and Recovery from other Stresses, which tests the Collaborative Care Model for primary care patients with opioid use disorder and co-occurring depression and/or post-traumatic stress disorder (PTSD). We defined being unhoused as not living in stable housing in the past 3 months and being unstably housed as living in stable housing but being worried or concerned about loss of housing in the next 3 months. We assessed differences in baseline characteristics across the housing groups using ANOVA for continuous variables and chi-squared tests for categorical and binary measures. Adjusted linear regression models were used to assess associations between housing status and mental health and substance use symptom severity scores.

**Results:**

Among the 797 patients randomized, 13% of the sample was currently unhoused, 24% was unstably housed, and 63% was stably housed. Individuals who were unhoused were on average younger, received less education, never married, and had not used prescribed medications for opioid use disorder (MOUD) in the past 30 days. The adjusted regression results showed that both being unhoused and being unstably housed were significantly associated with higher PTSD symptom severity, depression symptom severity, opioid use severity, and opioid overdose risk behaviors compared to being stably housed.

**Conclusion:**

Primary care patients with co-occurring disorders who were either unhoused or unstably housed have worse mental health and substance use symptom severity when compared with stably housed individuals. This suggests primary care providers should screen patients with co-occurring disorders not only for being unhoused but also for unstable housing. Addressing housing instability in primary care settings could lead to improved health outcomes and reduced healthcare costs.

**Trial registration:**

Clinicaltrials.gov NCT04559893, registered on January 8, 2021.

**Supplementary Information:**

The online version contains supplementary material available at 10.1186/s12875-025-02947-2.

## Background

Housing is a critical social determinant of health (SDOH) [1–4], and in January 2023, an estimated 650,000 people in the United States experienced homelessness [5]. Lack of stable housing is associated with worse physical and mental health outcomes, as well as increased mortality and healthcare costs [6, 7]. People experiencing homelessness (PEH), defined as those sleeping in a homeless shelter or other place not designed for long-term residential living (e.g., a tent, makeshift shelter, vehicle, or on the street) [8], have substantially higher rates of opioid overdose [9] and opioid overdose mortality than the general population [10]. Experiencing homelessness is a risk factor for opioid use disorder (OUD) and vice versa [11, 12]; and experiencing either homelessness or OUD is associated with a greater risk of having one or more mental health disorders [13]. For those with co-occurring OUD and mental health disorders (COD), experiencing homelessness exacerbates known difficulties getting treatment [14] for opioid use [15] and mental health disorders [16].

Despite growing knowledge of the health outcomes of PEH, less is known about the health of people experiencing unstable housing. Part of this knowledge gap is likely explained by the lack of standard definitions or measures of housing instability [2, 17, 18]. In late 2021, the Centers for Disease Control and Prevention introduced three ICD-10-CM Z codes for social determinants of health including housing instability [19]; however, difficulties remain with measuring unstable housing because the codes are used infrequently and have low sensitivity and specificity [20]. Capturing the nuance of housing-related problems is challenging because housing status exists on a continuum, and different studies define housing instability in different ways. For example, in some studies, the definition of unstable housing includes being actively unhoused [17, 18], whereas in others it does not [21]. Further complicating the assessment of housing status, a recent review of screening tools for housing status identified 31 relevant screening tools [22]. Without a standard definition and measure of unstable housing, it is challenging to understand the role it may play in health outcomes.

Despite the difficulties with defining unstable housing, evidence indicates that unstable housing makes treatment for OUD more challenging. Among people seeking treatment for OUD, those with housing instability were more likely to receive treatment in hospitals and rehabilitation centers and were less likely to receive medications for opioid use disorder (MOUD) [23]. Similarly, PEH are more likely to get inpatient and detoxification treatment for OUD than to receive MOUD [15]. This is concerning because MOUD, including buprenorphine and methadone, are evidence-based treatments for OUD that improve outcomes and are associated with fewer opioid overdoses and opioid-related acute care. Additionally, even when MOUD is provided in inpatient or residential settings, rates of linkage to treatment after discharge are low [24–26].

Increased utilization of primary care, particularly for those with housing instability and COD, is important for helping address complex health needs through better coordination and continuity of care [27]. Primary care can offer valuable services for PEH such as team-based, integrated care, co-located primary care, mental health, and social services, and outreach and care management [28]. However, these benefits may not reach patients with unstable housing if they are not identified. More research is needed to understand the characteristics of primary care patients with COD who are experiencing homelessness and unstable housing compared to those with stable housing. This study adds to the literature by examining the associations between mental health and substance use severity levels among primary care patients with COD reporting different housing statuses.

## Methods

### Study population and setting

The data for this paper are from a randomized controlled trial, Collaboration Leading to Addiction Treatment and Recovery from other Stresses (CLARO) that tests the effectiveness of collaborative care (CC) for adults with probable opioid use disorder and co-occurring depression and/or post-traumatic stress disorder (PTSD) [29]. The study included 4 health systems with 14 primary care clinics in New Mexico and 4 clinics in California. The clinics were located in both rural and urban areas and all served low-income populations.

Patients were recruited using several strategies to assess eligibility. The recruitment strategies were universal pre-screening on self-administered tablets in clinic waiting rooms, referrals from primary care providers or other clinic staff, outreach based on documented diagnoses in electronic health records (EHR), self-referral from patients on the CLARO website, or through participants recommending screening to family or friends (i.e. snowball sampling). Every patient who agreed was screened by study staff for probable OUD, depression and/or PTSD, and several additional eligibility criteria. Patients were only excluded from the study if they required immediate medical or psychiatric intervention. Further information on eligibility criteria can be found in the protocol manuscript [30]. A total of 797 patients were randomized between January 8, 2021, and December 5, 2023, and the baseline data for all randomized patients were included in these analyses. Baseline surveys were interviewer-administered prior to randomization.

### Measures

The *independent variable* was housing status with three categories: stably housed, unstably housed and unhoused. Housing status was assessed on the baseline survey with two questions: (1) In the past 3 months, have you been living in stable housing that you own, rent or stay in as part of a household? (2) Are you worried or concerned that in the next 3 months, you may (still) not have stable housing? [31] Stable housing was defined as a patient living in housing that they own, rent, or stay in as part of a household and do not worry about losing housing in the next 3 months. If a patient had not been living in stable housing in the past 3 months, we defined them as currently unhoused. If a patient had been living in stable housing but was worried or concerned that in the next 3 months they might not be living in stable housing, we defined them as unstably housed.

There were five *dependent variables* of interest, two for mental health severity and three for substance use severity. Mental health symptom severity was assessed by: (1) PTSD measured with the 20-item DSM-V PTSD checklist (PCL-5) [32] and (2) depression measured with the 9-item Patient Health Questionnaire (PHQ-9) [33]. Substance use severity was assessed by: (1) alcohol use severity measured with the 10-item Alcohol Use Disorder Identification Test (AUDIT) [34], (2) opioid use severity measured with the 7-item Patient Reported Outcomes Measurement Information System (PROMIS) [35], and (3) opioid overdose risk behaviors measured by the Opioid Overdose Risk Assessment tool (OORA) [36]. All measures were self-reported and the items within each measure were summed to create a total score that was used for these analyses.

Baseline patient characteristics served as *covariates* and included age in years, sex as it appears on current birth certificate (male; female), race and ethnicity categories (white, non-Hispanic; Hispanic, all races; and other), education categories (less than high school; high school or equivalent; and some college or more), marital status categories (never married; married or living with partner; and widowed, divorced or separated), health system affiliation (labeled 1–4), and prescribed MOUD use in the past 30 days (yes; no).

### Statistical analysis

Missingness in our baseline data was generally very low (less than 5%), and we followed best practices to handle missing data [37, 38]. In instances where responses were missing due to valid survey skip logic, we performed a series of logical imputations to impute zeros. Composite measures with item level missingness were scored following the measure scoring guideline’s algorithm. If guidance for handling missing items in a composite measure was not reported in the literature, we calculated a scaled sum. For all other missing items, we mean imputed values within treatment assignment groups and health care system. All data management and modelling were done in SAS 9.4 and bivariate comparisons and tables were done using Stata 17.

We examined the baseline characteristics of the sample across the three housing groups using ANOVA for continuous variables and chi-squared tests for categorical and binary measures. We then conducted adjusted linear regression models for each dependent variable, controlling for the aforementioned covariates, with results reported as regression coefficients and adjusted means with 95% confidence intervals to illustrate the size of the differences between the groups after adjustment [39]. Additionally, to reduce the probability of type I error with multiple testing of five outcomes, we performed a Benjamini-Hochberg correction with a false discovery rate (Q) = 0.05 on the joint F-tests to assess if findings were still statistically significance after adjustment for multiple testing [40].

## Results

### Patient characteristics

Table 1 shows descriptive statistics for the baseline characteristics among the total sample (*n* = 797) and across the three housing groups. 13% of the sample were currently unhoused, 24% were unstably housed, and 63% were stably housed. Individuals who were unhoused were on average younger, (*p* = 0.014), received less education (*p* = 0.02), and had never married (*p* = 0.001) when compared to those who were unstably housed and stably housed. Unhoused individuals were less likely to have been prescribed MOUD in the past 30 days (58.7%) compared to 72.3% and 77.8% of those who were unstably housed and stably housed, respectively (*p* < 0.001).

**Table 1:** Baseline patient characteristics

Characteristics	All (*N* = 797)	Currently Unhoused (*N* = 104)	Unstably Housed (*N* = 188)	Stably Housed (*N* = 505)	*P*-value
Age in years, mean (SD)	40.2 (11.9)	37.2 (11.2)	41.4 (11.6)	40.3 (12.0)	0.014
Sex^a,^ N (%)					0.107
Male	364 (45.7)	55 (52.9)	92 (48.9)	217 (43.0)	
Female	433 (54.3)	49 (47.1)	96 (51.1)	288 (57.0)	
Race and Ethnicity, N (%)					0.082
White, non-Hispanic	187 (23.5)	25 (24.0)	45 (23.9)	117 (23.2)	
Hispanic, all races	543 (68.1)	63 (60.6)	131 (69.7)	349 (69.1)	
Other^b^	67 (8.4)	16 (15.4)	12 (6.4)	39 (7.7)	
Education, N (%)					0.020
Less than high school	243 (30.5)	38 (36.5)	61 (32.4)	144 (28.5)	
High school or equivalent	229 (28.7)	35 (33.7)	40 (21.3)	154 (30.5)	
Some college or more	325 (40.8)	31 (29.8)	87 (46.3)	207 (41.0)	
Marital status, N (%)					0.001
Never married	288 (36.1)	53 (51.0)	69 (36.7)	166 (32.9)	
Married/living with partner	294 (36.9)	22 (21.2)	64 (34.0)	208 (41.2)	
Widowed/divorced/separated	215 (27.0)	29 (27.9)	55 (29.3)	131 (25.9)	
Health system, N (%)					< 0.001
System 1	381 (47.8)	51 (49.0)	92 (48.9)	238 (47.1)	
System 2	35 (4.4)	4 (3.8)	2 (1.1)	29 (5.7)	
System 3	321 (40.3)	32 (30.8)	79 (42.0)	210 (41.6)	
System 4	60 (7.5)	17 (16.3)	15 (8.0)	28 (5.5)	
Prior MOUD^c^, N (%)					< 0.001
No	207 (26.0)	43 (41.3)	52 (27.7)	112 (22.2)	
Yes	590 (74.0)	61 (58.7)	136 (72.3)	393 (77.8)	

^a^As it appears on current birth certificate

^b^Includes more than one race and non-Hispanic

^c^Prior use in the past 30 days, all was prescribed

### Adjusted associations between housing groups

Table 2 shows the multivariable linear regression results adjusting for age, sex, race and ethnicity, education, marital status, prescribed MOUD, and health system with betas and 95% confidence intervals. For mental health severity outcome measures, being unhoused was significantly associated with both higher PTSD symptom severity (β = 6.0, [2.3–9.8]) and higher depression symptom severity (β = 1.3, [0.1–2.5]) compared to those who were stably housed. Being unstably housed was also significantly associated with both higher PTSD symptom severity (β = 7.0, [4.1–9.9]) and higher depression symptom severity (β = 1.8, [0.8–2.7]) compared to those who were stably housed.

**Table 2:** Multivariable linear regression results for housing status

Dependent Variable		Currently Unhoused		Unstably Housed		*R* ^2^	*P*-Value
		β-coefficient	95% Confidence Interval	β-coefficient	95% Confidence Interval		
Mental Health Severity	PTSD symptom severity	6.0	2.3–9.8	7.0	4.1–9.9	0.051	< 0.001
	Depression symptom severity	1.3	0.1–2.5	1.8	0.8–2.7	0.062	< 0.001
Substance Use Severity	Alcohol use severity	-0.8	-2.8–1.1	1.4	-0.1–2.9	0.05	0.075
	Opioid use severity	5.1	3.4–6.8	3.8	2.5–5.1	0.168	< 0.001
	Opioid overdose risk behaviors	3.7	2.4–4.9	2.3	1.4–3.3	0.181	< 0.001

PTSD symptom severity is only calculated for patients who have had a traumatic event (*n* = 736)

Models are adjusting for age, sex, race/ethnicity, education, marital status, prior MOUD, health system

The stably housed group is the reference

For substance use severity outcome measures, being unhoused was significantly associated with both higher opioid use severity (β = 5.1, [3.4–6.8]) and higher opioid overdose risk behaviors (β = 3.7, [2.4–4.9]) compared to those who were stably housed. Being unstably housed was significantly associated with both higher opioid use severity (β = 3.8, [2.5–5.1]) and higher opioid overdose risk behaviors (β = 2.3, [1.4–3.3]) compared to those who were stably housed. All findings were confirmed after adjustment for multiple testing with the exception of alcohol use severity, where we found no evidence of an association between housing status and alcohol use severity.

**Fig. 1:**
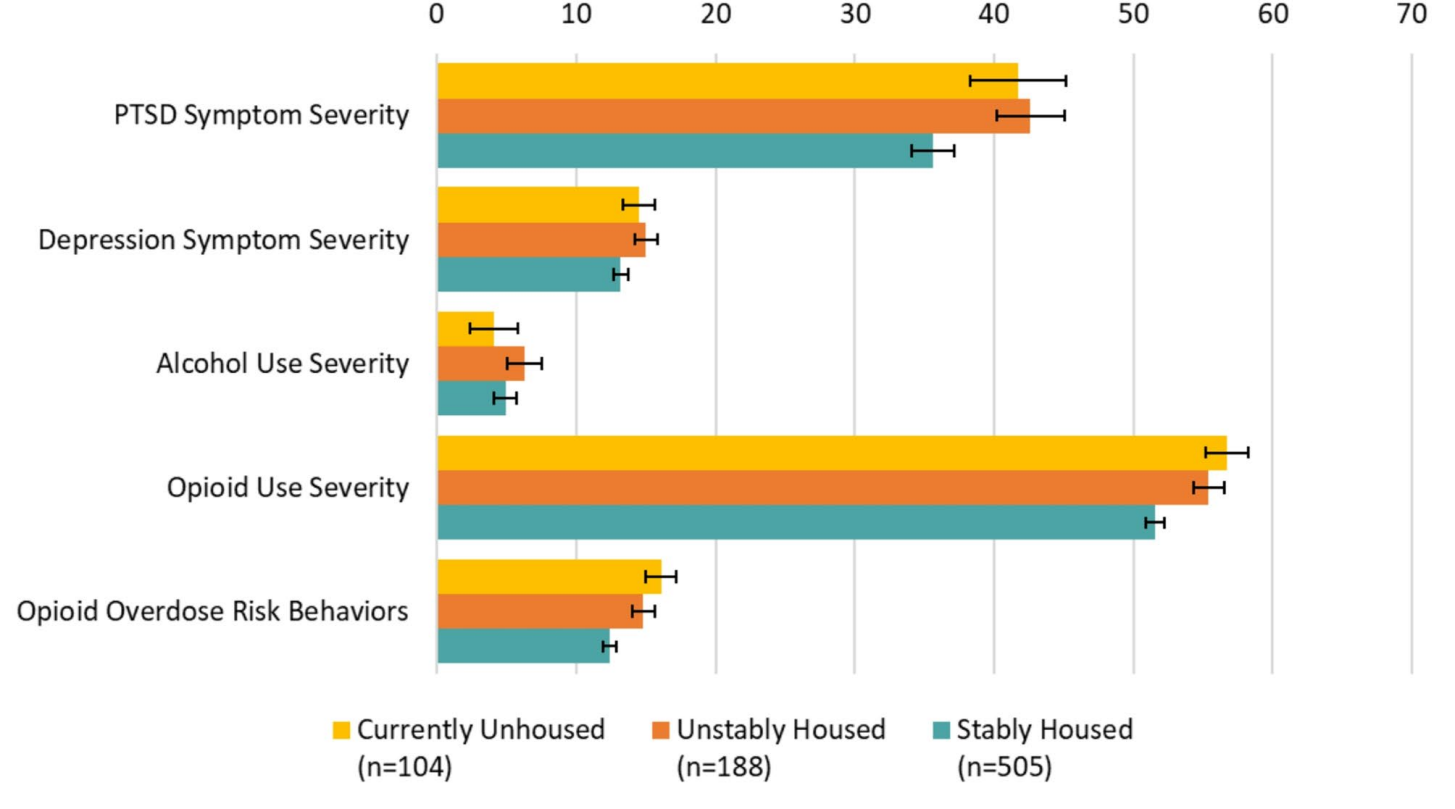
Adjusted means by housing status with 95% confidence intervals

Figure 1 shows the adjusted means from our multivariable linear regression models, with error bars representing 95% confidence intervals, for all dependent variables to illustrate the size of the differences between the housing groups for each variable. Adjusted means for PTSD symptom severity were significantly lower among the stably housed (35.6, [34.1–37.1]) compared to both the unstably housed (42.6, [40.2–45.1]) and the unhoused (41.7, [38.2–45.1]). Adjusted means for depression symptom severity were significantly higher for the unstably housed (15.0, [14.2–15.8]) compared to the stably housed (13.2, [12.7–13.7]).

For the substance use measures, adjusted mean scores were highest among those who were unhoused for the opioid use severity (56.7, [55.2–58.2]) and opioid overdose risk behaviors measures (16.1, [15.0-17.2]), followed by those who were unstably housed (55.4, [54.3–56.5] for severity and 14.8, [13.9–15.6] for overdose risk), and lowest among the stably housed (51.6, [50.9–52.3] for severity and 12.4, [11.9–12.9] for overdose risk).

## Discussion

This study finds there are meaningful clinical differences between primary care patients with COD who are unhoused or unstably housed compared to those who are stably housed. Patients who are unhoused are more likely to be younger, have received less education, have never married, and have not taken prescribed MOUD in the past 30 days than both unstably and stably housed patients. Both unhoused and unstably housed individuals have higher mental health and substance use symptom severity than stably housed individuals. Importantly, our findings show that health risks are higher for both the unhoused and the unstably housed compared to the stably housed.

Our results add to the evidence around the relationship between housing status and mental health and substance use severity among individuals with COD. Past research has shown that PEH have more severe mental health and substance use symptom severity compared to stably housed individuals [1]; our study indicates that this disparity extends to the unstably housed, that is, those who are currently housed but worry about losing their housing in the near future [41]. Individuals with unstable housing are similarly at risk of complex health problems as PEH, demonstrated by a study that found that a greater percentage of vulnerably housed individuals reported having COD relative to PEH [27].

The findings of our study do not undermine the complexity and health risks for PEH. Rather, these findings highlight how individuals who are unstably housed may be struggling and need additional support. Housing stress, even when one is housed, can impact mental health and substance use severity [41]. PEH may access services through shelters that improve mental health and substance use problems and reduce daily stress; however, people who are unstably housed are less likely to go to shelters and access these services [42]. Furthermore, there are outreach interventions to improve behavioral health for PEH, such as Street Medicine [43], that are unlikely to reach those who are housed but worried about losing housing. The unstably housed population may lack access to services targeting their housing status, and therefore, efforts to identify unstably housed individuals and their needs in different settings are important.

Our results suggest that patients with COD would benefit from screening for both homelessness and housing instability in a primary care setting. Primary care is often a patient’s main point of interaction with the healthcare system, allowing for the development of longer-term relationships between the patient and clinician [44]. A majority of patients have indicated comfort with screening for and documenting SDOH in primary care clinical settings, particularly if they trust their clinician and/or had previously received assistance with SDOH [22, 45]. Furthermore, widespread adoption of EHR technology in primary care facilitates documentation of ICD-10-CM Z codes to track SDOH, and also benefits from buy-in from primary care providers, leadership, and clinic staff [42].

Although research indicates providers are comfortable screening for SDOH [46], screening for housing instability in primary care remains low [47], indicating that clinicians face barriers to doing so. Research suggests that team-based approaches, screening patients for their needs, being prepared to refer patients to community-based resources, and embedding these elements into the clinical workflow are key for addressing SDOH, including housing status [46], but not all primary care practices have sufficient resources to do so. Newly introduced ICD-10-CM Z codes can facilitate documentation of housing instability and inform both clinical practice and population health research, but these codes are largely non-billable [19]. One bright spot is that screening for housing instability and uptake of ICD-10-CM Z code documentation is higher in physician practices paid via alternative delivery and payment models, including Federally Qualified Health Centers, bundled payment participants, primary care improvement models, and Medicaid accountable care organizations [47]. This indicates that successful implementation of screening for SDOH hinges on payment models that will support primary care teams for this effort.

Additional research is needed to optimize screening for social needs in primary care settings, including understanding contextual factors like the structure of healthcare financing that make screening effective and feasible. Importantly, research is needed to identify if screening for social needs like housing instability leads to services and improves health outcomes.

Some limitations should be noted. First, there is no standard measure of unstable housing [2, 17, 18], and the measurement of unstable housing in this paper may be different from how other studies operationalize the concept. Next, this study comes from a clinical trial at primary care clinics in New Mexico and California. The results may not be generalizable to other areas of the United States, other countries, or other clinical settings. Due to the nature of patients in this study already having contact with primary care, the population in this sample may be more engaged with healthcare, MOUD, and mental health treatment. Therefore, the study is not representative of people who are less engaged with the health care system. Next, adjustment for additional socioeconomic factors, such as income and employment status, was not possible, as these variables were not collected in the survey. Finally, the conclusions are limited to this population with COD, but these patients are often not identified. According to the 2023 National Survey on Drug Use and Health, about 2 in 5 adults with co-occurring mental illness and substance use disorders did not receive treatment for either condition in the past year [48]. Therefore, clinics that are not implementing universal screening for COD might consider screening all patients for unstable housing, in order to ensure that no patients are missed. Despite these limitations, the major strength of this paper is the exploration of clinical characteristics by housing status among a complex patient population of people with COD.

## Conclusions

The findings of this study reveal that patients with COD experiencing unstable housing, defined as patients who self-report being worried about not having stable housing in the next 3 months, are at increased risk for substance use and mental health symptom severity at similar levels as PEH. People experiencing unstable housing may not be identified as such, and therefore, may not receive needed services to improve their social determinants of health. Primary care may be an ideal setting to increase identification of unstable housing and connect patients who have complex health and social problems to needed care.

## Supplementary Information

Supplementary Material 1.

## Data Availability

The datasets generated and/or analyzed during the current study are not currently publicly available, but trial data will be made available via the NIMH Data Archive pending NIH approval of data structures. Interested parties can search for Project Number UF1MH121954-01. De-identified data will be made available from the corresponding author on reasonable request.

## Abbreviations

PTSD: Post-traumatic stress disorder
MOUD: Medications for opioid use disorder
OUD: Opioid use disorder
COD: Co-occurring disorders
PEH: People experiencing homelessness
CLARO: Collaboration Leading to Addiction Treatment and Recovery from other Stresses
CC: Collaborative care
PCL-5: DSM-V symptoms of PTSD checklist
PHQ-9: 9-item Patient Health Questionnaire
AUDIT: Alcohol Use Disorder Identification Test
PROMIS: Patient Reported Outcomes Measurement Information System
OORA: Opioid Overdose Risk Assessment tool
SDOH: Social determinant of health
EHR: Electronic health records
