# Supplementary material for: Association between housing status and mental health and substance use severity among individuals with opioid use disorder and co-occurring depression and/or PTSD
Source: BMC Prim Care. 2025 Aug 8;26:250. doi: 10.1186/s12875-025-02947-2 (PMC12333051; doi:10.1186/s12875-025-02947-2)
Supplement: Supplementary file 1 — Supplementary Material 1. [file 12875_2025_2947_MOESM1_ESM.docx]

**BASELINE INTERVIEW**

**P1. Language [lang]**

English [1]

Español [2]

**P2. Today’s date:** Month: __ __ Day: __ __ Year: __ __ __ __ [interview date]

**P3. Clinic name: ___________________ [org]**

[Page break]

**Thank you for taking the time to complete this research interview. All of your answers are confidential. Remember that you can skip questions or stop the interview at any time.**

**We'll start by getting some basic background information from you. Do you have any questions before we begin?**

**1. How old are you today?**

______________ years [RANGE: 18 - 120] [variable name = age]

☐ DON’T KNOW [888]

☐ REFUSE [999]

**What is your full birthday? [No variable name?]**

Month: __ __ Day: __ __ Year: __ __ __ __

☐ DON’T KNOW [888]

☐ REFUSE [999]

*(Part of Identification Section, moved for continuity)*

**2. What is your sex, as currently listed on your birth certificate? [sex]**

Male [1]

Female [2]

Gender-neutral sex designation (X) [3]

☐ DON’T KNOW [888]

☐ REFUSE [999]

**3. Do you consider yourself to be Hispanic, Latino, or of Spanish origin? [Hispanic]**

☐ Yes [1]

☐ No [2]

☐ DON’T KNOW [888]

☐ REFUSE [999]

**4. What race or races do you consider yourself to be? [CHECK ALL THAT APPLY] [**race]

☐ American Indian or Alaska Native [race___1] [1]

☐ Asian [race___2] [2]

☐ Native Hawaiian or Pacific Islander [race___3] [3]

☐ Black or African American [race___4] [4]

☐ White [race___5] [5]

☐ Other [race___6] [6]

☐ DON’T KNOW [race___888] [888]

☐ REFUSE [race___999] []

**IF OTHER, what race(s)? __________________** [race_other]

**5. What is the highest grade or year of school you have completed? [DO NOT READ THE RESPONSE OPTIONS TO THE PARTICIPANT]**

[education]

☐ NO SCHOOLING COMPLETED/KINDERGARTEN ONLY [1]

☐ 1ST GRADE [2]

☐ 2ND GRADE [3]

☐ 3RD GRADE [4]

☐ 4TH GRADE [5]

☐ 5TH GRADE [6]

☐ 6TH GRADE [7]

☐ 7TH GRADE [8]

☐ 8TH GRADE [9]

☐ 9TH GRADE [10]

☐ 10TH GRADE [11]

☐ 11TH GRADE [12]

☐ 12TH GRADE, NO DIPLOMA [13]

☐ HIGH SCHOOL GRADUATE [14]

☐ GED CERTIFICATE [15]

☐ SOME COLLEGE CREDIT, BUT NO DEGREE [16]

☐ OCCUPATIONAL, TECHNICAL OR VOCATIONAL CERTIFICATE [17]

☐ ASSOCIATE'S DEGREE (AA, AS) [18]

☐ BACHELOR’S DEGREE (BA, BS) [19]

☐ MASTER’S DEGREE (MA, MS, M. ENG, M. ED, MSW, MBA) [20]

☐ ACADEMIC DOCTORATE DEGREE (PHD, EDD) [21]

☐ PROFESSIONAL DEGREE BEYOND A BACHELOR’S DEGREE (MD, DDS, DVM, LLB, JD) [22]

☐ DON'T KNOW [888]

☐ REFUSE [999]

**6.** **Are you now married, widowed, divorced, separated, never married, or living with a partner?** [DO NOT READ THE RESPONSE OPTIONS TO THE PARTICIPANT] [married]

☐ MARRIED [1]

☐ NEVER MARRIED [2]

☐ SEPARATED [3]

☐ DIVORCED [4]

☐ WIDOWED [5]

☐ LIVING WITH PARTNER [6]

☐ DON’T KNOW [888]

☐ REFUSE [999]

**7. What is your five-digit ZIP code? This should be for the primary place where you live or stay.**

__ __ __ __ __ [RANGE: 00000 - 99999] [Variable Name = c_zip]

☐ DON’T KNOW [888]

☐ REFUSE [999]

**Pain** (PEG)

**Let’s turn to questions about your health. Now think about pain you have had during the past 7 days, from [DATEFILL] up to and including today.**

**1. What number best describes your pain, on average?** [c_pain1]

| 🞎  0 [0] | 🞎  1 [1] | 🞎  2 [2] | 🞎  3 [3] | 🞎  4 [4] | 🞎  5 [5] | 🞎  6 [6] | 🞎  7 [7] | 🞎  8 [8] | 🞎  9 [9] | 🞎  10 [10] |
| --- | --- | --- | --- | --- | --- | --- | --- | --- | --- | --- |
| No pain |  |  |  |  |  |  |  |  |  | Pain  as bad as you can imagine |

☐ DON’T KNOW [888]

☐ REFUSE [999]

**2. During the past 7 days, what number best describes how pain has interfered with your enjoyment of life?** [c_pain2]

| 🞎  0[0] | 🞎  1[1] | 🞎  2[2] | 🞎  3[3] | 🞎  4[4] | 🞎  5[5] | 🞎  6[6] | 🞎  7[7] | 🞎  8[8] | 🞎  9[9] | 🞎  10[10] |
| --- | --- | --- | --- | --- | --- | --- | --- | --- | --- | --- |
| Does not interfere |  |  |  |  |  |  |  |  |  | Completely interferes |

☐ DON’T KNOW [888]

☐ REFUSE [999]

**3. During the past 7 days, what number best describes how pain has interfered with your general activity?** [c_pain3]

| 🞎  0[0] | 🞎  1[1] | 🞎  2[2] | 🞎  3[3] | 🞎  4[4] | 🞎  5[5] | 🞎  6[6] | 🞎  7[7] | 🞎  8[8] | 🞎  9[9] | 🞎  10[10] |
| --- | --- | --- | --- | --- | --- | --- | --- | --- | --- | --- |
| Does not interfere |  |  |  |  |  |  |  |  |  | Completely interferes |

☐ DON’T KNOW [888]

☐ REFUSE [999]

**I am going to ask you some questions about using different drugs. All of your answers are confidential, and we will not share this information with anyone outside of the research project.**

**Alcohol use severity (AUDIT)**

**Now I am going to ask you some questions about your use of alcoholic beverages during the past 3 months, from [DATEFILL] up to and including today. An alcoholic drink means one beer (12 oz.), one small glass of wine (5 oz.), or one mixed drink containing one shot (1.5 oz.) of hard liquor such as vodka, gin, tequila, rum, or whisky.**

**1. How often do you have a drink containing alcohol?** [c_audit1]

☐ Never [0]

☐ Once a month or less [1]

☐ Monthly [2]

☐ Weekly [3]

☐ 2-3 times a week [4]

☐ 4-6 times a week [5]

☐ Daily [6]

☐ DON’T KNOW [888]

☐ REFUSE [999]

[If 1 = NEVER/REFUSE, skip to Substance Use (NDSUH)]

**2. How many drinks containing alcohol do you have on a typical day when you are drinking?** [c_audit2]

☐ 1 drink [0]

☐ 2 drinks [1]

☐ 3 drinks [2]

☐ 4 drinks [3]

☐ 5-6 drinks [4]

☐ 7-9 drinks [5]

☐ 10 or more drinks [6]

☐ DON’T KNOW [888]

☐ REFUSE [999]

[IF sex = MALE and age ≤ 65, display Question 3a]

[ELSE, display Question 3b]

**3a. How often do you have 5 or more drinks on one occasion?** [c_audit3a]

☐ Never [0]

☐ Less than monthly [1]

☐ Monthly [2]

☐ Weekly [3]

☐ 2-3 times a week [4]

☐ 4-6 times a week [5]

☐ Daily [6]

☐ DON’T KNOW [888]

☐ REFUSE [999]

**3b. How often do you have 4 or more drinks on one occasion?** [c_audit3b]

☐ Never [0]

☐ Less than monthly [1]

☐ Monthly [2]

☐ Weekly [3]

☐ 2-3 times a week [4]

☐ 4-6 times a week [5]

☐ Daily [6]

☐ DON’T KNOW [888]

☐ REFUSE [999]

[If 2 = 1 DRINK/REFUSE AND 3 = NEVER/REFUSE, skip to Substance Use (NDSUH)]

**How often during the past 3 months, from [DATEFILL] up to and including today, have you…**

|  | **Never** | **Less than monthly** | **Monthly** | **Weekly** | **Daily or almost daily** | **DON’T KNOW** | **REFUSE** |
| --- | --- | --- | --- | --- | --- | --- | --- |
| **4. …found that you were not able to stop drinking once you started?** [c_audit4] | ☐[0] | ☐ [1] | ☐ [2] | ☐ [3] | ☐ [4] | ☐ [888] | ☐ [999] |
| **5. …failed to do what was expected of you because of drinking?** [c_audit5] | ☐[0] | ☐ [1] | ☐ [2] | ☐ [3] | ☐ [4] | ☐ [888] | ☐ [999] |
| **6. …needed a first drink in the morning to get yourself going after a heavy drinking session?** [c_audit6] | ☐ [0] | ☐ [1] | ☐ [2] | ☐ [3] | ☐ [4] | ☐ [888] | ☐ [999] |
| **7. …had a feeling of guilt or remorse after drinking?** [c_audit7] | ☐ [0] | ☐ [1] | ☐ [2] | ☐ [3] | ☐ [4] | ☐ [888] | ☐ [999] |
| **8. …been unable to remember what happened the night before because you had been drinking?** [c_audit8] | ☐ [0] | ☐ [1] | ☐ [2] | ☐ [3] | ☐ [4] | ☐ [888] | ☐ [999] |

**9. Have you or someone else been injured because of your drinking?** [c_audit9]

☐ No[0]

☐ Yes, but not in the last 3 months [2]

☐ Yes, during the last 3 months [4]

☐ DON’T KNOW [888]

☐ REFUSE [999]

**10. Has a relative, friend, doctor, or other health worker been concerned about your drinking and suggested you cut down?** [c_audit10]

☐ No[0]

☐ Yes, but not in the last 3 months [2]

☐ Yes, during the last 3 months [4]

☐ DON’T KNOW [888]

☐ REFUSE [999]

**Substance Use (National Survey on Drug Use and Health)**

**1. Think specifically about the past 30 days, from [DATEFILL] up to and including today. During the past 30 days, on how many days did you use illegal or street drugs, or use prescription drugs in any way a doctor did not direct you to use them? Do not include over-the-counter pills, alcohol, or marijuana when counting the number of days.**

_____ days [RANGE: 0 - 30] [c_nsduh1]

☐ DON’T KNOW [888]

☐ REFUSE [999]

**2. In the past 30 days, did you take any opioid prescription pain pills, even just once (for example: Oxycodone, Percocet, Vicodin)? I am not asking about over-the-counter pills.** [c_nsduh2]

☐ Yes [1]

☐ No [0]

☐ DON’T KNOW [888]

☐ REFUSE [999]

[If 2 = NO/DK/REFUSE, skip to question 3]

**2a. In the past 30 days, did you take any opioid prescription pain pills that were not prescribed for you?** [c_nsduh2a]

☐ Yes [1]

☐ No [0]

☐ DON’T KNOW [888]

☐ REFUSE [999]

**2b. In the past 30 days, have you ever taken more opioid prescription pain pills than your provider told you to?** [c_nsduh2b]

☐ Yes [1]

☐ No [0]

☐ DON’T KNOW [888]

☐ REFUSE [999]

**3. In the past 30 days, did you use heroin or fentanyl, even just once?** [c_nsduh3]

☐ Yes [1]

☐ No [0]

☐ DON’T KNOW [888]

☐ REFUSE [999]

[If 2a, 2b, AND 3 = NO/DK/REFUSE, skip to question 5]

**4. During the past 30 days, on how many days did you use heroin, fentanyl, and/or use opioid prescription pain pills in any way a health care provider did not direct you to use them?** [c_nsduh4]

_____ days [RANGE: 0 - 30]

☐ DON’T KNOW [888]

☐ REFUSE [999]

**5. Think specifically about the past 30 days, from [DATEFILL] up to and including today. During the past 30 days, on how many days did you use cocaine or crack? This includes all the different forms of cocaine such as powder, “crack,” free base, and coca paste.**

_____ days [RANGE: 0 - 30] [c_nsduh5]

☐ DON’T KNOW [888]

☐ REFUSE [999]

**6. During the past 30 days, on how many days did you use methamphetamine or other stimulants (in any way a health care provider did not direct you to use them)? Methamphetamine is also called crystal meth or speed. Other stimulants are pills that people take for attention deficit disorders (like Adderall, Ritalin), to lose weight (like Tenuate), or to stay awake (like Provigil) – if you used them in any way a provider did not direct you to use them. I am not asking about over-the-counter pills.**

_____ days [RANGE: 0 - 30] [c_nsduh6]

☐ DON’T KNOW [888]

☐ REFUSE [999]

**7. During the past 30 days, on how many days did you use sedatives or tranquilizers (in any way a health care provider did not direct you to use them)? Sedatives are also called “downers” or “sleeping pills” (like Ambien, Lunesta, or Phenobarbital). Tranquilizers relax people or help muscle spasms (like Xanax, Ativan, Klonopin, or Valium) – if you used them in any way a provider did not direct you to use them. I am not asking about over-the-counter pills.**

_____ days [RANGE: 0 - 30] [c_nsduh7]

☐ DON’T KNOW [888]

☐ REFUSE [999]

**Opioid use severity (PROMIS – Substance Use – Short Form)**

**The following questions ask about your use of opioids (e.g., Heroin, Morphine, Dilaudid, Demerol, Oxycontin, oxy, codeine (Tylenol 2,3,4), Percocet, Vicodin, Fentanyl, etc.) other than your prescribed Buprenorphine. In the past 30 days, from [DATEFILL] up to and including today…**

**1. I felt that my opioid use was out of control.** [c_promis1]

☐ Not at all [1]

☐ A little bit [2]

☐ Somewhat [3]

☐ Quite a bit [4]

☐ Very much [5]

☐ DON’T KNOW [888]

☐ REFUSE [999]

|  | **Never** | **Rarely** | **Sometimes** | **Often** | **Almost always** | **DON’T KNOW** | **REFUSE** |
| --- | --- | --- | --- | --- | --- | --- | --- |
| **2. My desire to use opioids seemed overpowering.** [c_promis2] | ☐ [1] | ☐ [2] | ☐ [3] | ☐ [4] | ☐ [5] | ☐ [888] | ☐ [999] |
| **3. Opioids were the only thing I could think about.** [c_promis3] | ☐ [1] | ☐ [2] | ☐ [3] | ☐ [4] | ☐ [5] | ☐ [888] | ☐ [999] |
| **4. My opioid use caused problems with people close to me.** [c_promis4] | ☐ [1] | ☐ [2] | ☐ [3] | ☐ [4] | ☐ [5] | ☐ [888] | ☐ [999] |

**5. I have an opioid use problem.** [c_promis5]

☐ Not at all [1]

☐ A little bit [2]

☐ Somewhat [3]

☐ Quite a bit [4]

☐ Very much [5]

☐ DON’T KNOW [888]

☐ REFUSE [999]

**6. I craved opioids.** [c_promis6]

☐ Never [1]

☐ Rarely [2]

☐ Sometimes [3]

☐ Often [4]

☐ Almost always [5]

☐ DON’T KNOW [888]

☐ REFUSE [999]

**7. I spent a lot of time using opioids.** [c_promis7]

☐ Never [1]

☐ Rarely [2]

☐ Sometimes [3]

☐ Often [4]

☐ Almost always [5]

☐ DON’T KNOW [888]

☐ REFUSE [999]

**Overdose risk level (****Opioid Overdose Risk Assessment)**

**In the past 30 days, from [DATEFILL] up to and including today, how often…**

|  | **Never** | **Rarely** | **Sometimes** | **Often** | **Very often** | **DON’T KNOW** | **REFUSE** |
| --- | --- | --- | --- | --- | --- | --- | --- |
| **1. … have you used an opioid (such as heroin, fentanyl, or painkillers) when nobody else was around?** [c_oora1] | ☐ [0] | ☐ [1] | ☐ [2] | ☐[3] | ☐ [4] | ☐ [888] | ☐ [999] |
| **2. … have you used opioids (such as heroin, fentanyl, or painkillers) in a place where you don’t usually use them?** [c_oora2] | ☐ [0] | ☐ [1] | ☐ [2] | ☐ [3] | ☐ [4] | ☐ [888] | ☐ [999] |
| **3. … did you drink alcohol within 2 hours before or after using an opioid (such as heroin, fentanyl, or painkillers)?** [c_oora3] | ☐ [0] | ☐ [1] | ☐ [2] | ☐ [3] | ☐ [4] | ☐ [888] | ☐ [999] |
| **4. … did you take sedatives (such as Xanax) within 2 hours before or after using an opioid (such as, heroin, fentanyl, or painkillers)?** [c_oora4] | ☐ [0] | ☐ [1] | ☐ [2] | ☐ [3] | ☐ [4] | ☐ [888] | ☐ [999] |
| **5. … did you use heroin or fentanyl within 2 hours before or after using another opioid (such as painkillers)?** [c_oora5] | ☐ [0] | ☐ [1] | ☐ [2] | ☐ [3] | ☐ [4] | ☐ [888] | ☐ [999] |

**6. In the past 30 days, from [DATEFILL] up to and including today, how often did you use uppers (such as crack, cocaine, crystal/meth) within 2 hours before or after using an opioid (such as, heroin, fentanyl, or painkillers)?** [c_oora6]

☐ Never [0]

☐ Once [1]

☐ More than once [2]

☐ DON’T KNOW [888]

☐ REFUSE [999]

**In the past 30 days, how often…**

|  | **Never** | **Rarely** | **Sometimes** | **Often** | **Very often** | **DON’T KNOW** | **REFUSE** |
| --- | --- | --- | --- | --- | --- | --- | --- |
| **7. … have you increased the amount of an opioid (such as heroin, fentanyl, or painkillers) you used to more than you usually use?** [c_oora7] | ☐ [0] | ☐ [1] | ☐ [2] | ☐ [3] | ☐ [4] | ☐ [888] | ☐ [999] |
| **8. …** **have you snorted any drugs? (This includes huffing paint or chemicals)** [c_oora8] | ☐ [0] | ☐ [1] | ☐ [2] | ☐ [3] | ☐ [4] | ☐ [888] | ☐ [999] |
| **9. … have you injected any drugs?** [c_oora9] | ☐ [0] | ☐ [1] | ☐ [2] | ☐ [3] | ☐ [4] | ☐ [888] | ☐ [999] |

**History of overdoses (Overdose Baseline Questionnaire from** [**naloxoneinfo.org**](http://www.naloxoneinfo.org)**)**

**Now for these questions, I am asking you about opioid overdoses. By overdose I mean someone who collapses, has blue skin color, convulsions, difficulty breathing, loses consciousness, cannot be woken up, or has a heart attack or dies while using drugs.**

**1. Have you ever overdosed in your life?** [c_obq1]

□ Yes [1]

□ No [0]

☐ DON’T KNOW [888]

☐ REFUSE [999]

[If 1 = NO/DK/REFUSE, skip to Depression (PHQ-9)]

**2. Have you overdosed in the past 3 months, from [datefill_3mo] up to and including today?** [c_obq2]

□ Yes [1]

□ No [0]

☐ DON’T KNOW [888]

☐ REFUSE [999]

[If 2 = NO/DK/REFUSE, skip to Depression (PHQ-9)]

**3. How many times (in the past 3 months)? [Range = 0-100]** [c_obq3]

_____ times

☐ DON’T KNOW [888]

☐ REFUSE [999]

**These questions are about what happened at your last overdose.**

**4. What were the drugs you were using at the time of your last overdose?** [c_obq4], derived variable is [c_obq4_dv]

□ Benzos (like Xanax, Ativan, Klonopin, or Valium) [c_obq4___1] [1]

□ Opiates (like heroin, fentanyl, Oxycodone, Percocet, or Vicodin) [c_obq4___2] [2]

□ Stimulants or uppers (like methamphetamine/crystal meth/speed) [c_obq4___3] [3]

□ Methadone[c_obq4___4] [4]

☐ Alcohol[c_obq4___5] [5]

☐ Other[c_obq4___6] [6]

☐ DON’T KNOW[c_obq4___888] [7]

☐ REFUSE[c_obq4___999] [8]

**IF OTHER, which drug(s)?** [c_obq4___other] **____________________________________________________**

**5. Did someone call for an ambulance or take you to the hospital?** [c_obq5]

□ Yes [1]

□ No [0]

☐ DON’T KNOW [888]

☐ REFUSE [999]

**6. Did you receive professional medical help (from a doctor, nurse, or EMS)?** [c_obq6]

□ Yes [1]

□ No [0]

☐ DON’T KNOW [888]

☐ REFUSE [999]

**7. Were you given Naloxone/Narcan?** [c_obq7]

□ Yes [1]

□ No [0]

☐ DON’T KNOW [888]

☐ REFUSE [999]

**These next questions ask about your mental health. All of your answers are confidential, and we will not share this information with anyone outside of the research project.**

**Have more than 3 days passed since the Eligibility screener was administered?** [c_phq_3days]

□ Yes [1]

□ No [0]

[If YES, continue with Depression (PHQ-9 full measure)]

[If NO, skip to Depression (PHQ item 9 only)]

**Depression (PHQ-9 full measure)**

**Over the last 2 weeks, from [DATEFILL] up to and including today, how often have you been bothered by any of the following problems?**

|  | **Not at all** | **Several days** | **More than half the days** | **Nearly every day** | **DON’T KNOW** | **REFUSE** |
| --- | --- | --- | --- | --- | --- | --- |
| **1. Little interest or pleasure in doing things** [c_phq1] | ☐ [0] | ☐[1] | ☐ [2] | ☐ [3] | ☐ [888] | ☐ [999] |
| **2. Feeling down, depressed, or hopeless** [c_phq2] | ☐ [0] | ☐ [1] | ☐ [2] | ☐ [3] | ☐ [888] | ☐ [999] |
| **3. Trouble falling or staying asleep, or sleeping too much** [c_phq3] | ☐ [0] | ☐ [1] | ☐ [2] | ☐ [3] | ☐ [888] | ☐ [999] |
| **4. Feeling tired or having little energy** [c_phq4] | ☐ [0] | ☐ [1] | ☐ [2] | ☐ [3] | ☐ [888] | ☐ [999] |
| **5. Poor appetite or overeating** [c_phq5] | ☐ [0] | ☐ [1] | ☐ [2] | ☐ [3] | ☐ [888] | ☐ [999] |
| **6. Feeling bad about yourself – or that you are a failure or have let yourself or your family down** [c_phq6] | ☐ [0] | ☐ [1] | ☐ [2] | ☐ [3] | ☐ [888] | ☐ [999] |
| **7. Trouble concentrating on things, such as reading the newspaper or watching television** [c_phq7] | ☐ [0] | ☐ [1] | ☐ [2] | ☐ [3] | ☐ [888] | ☐ [999] |
| **8. Moving or speaking so slowly that other people could have noticed. Or the opposite – being so fidgety or restless that you have been moving around a lot more than usual** [c_phq8] | ☐ [0] | ☐ [1] | ☐ [2] | ☐ [3] | ☐ [888] | ☐ [999] |
| **9. Thoughts that you would be better off dead, or of hurting yourself** [c_phq9] | □ [0] | □ [1] | □ [2] | □ [3] | □ [888] | □ [999] |

[Skip next to Suicidal Ideation (CSSRS)]

**Depression (PHQ item 9 only)**

**The next questions ask about thoughts, feelings, or actions related to suicide or hurting oneself. Many primary care patients have these experiences. So we ask everyone about them. Over the last 2 weeks, from [DATEFILL] up to and including today, how often have you been bothered by thoughts that you would be better off dead, or of hurting yourself?**

□ Not at all [0]

☐ Several days [1]

☐ More than half the days [2]

☐ Nearly every day [3]

☐ DON’T KNOW [888]

☐ REFUSE [999]

**Suicidal ideation (Columbia Suicide Severity Rating Scale)**

**1. In the past 30 days, from [DATEFILL] up to and including today, have you wished you were dead or wished you could go to sleep and not wake up?** [c_cssr1]

□ Yes [1]

□ No [0]

☐ DON’T KNOW [888]

☐ REFUSE [999]

**2. In the past 30 days, have you had any actual thoughts of killing yourself?** [c_cssr2]

□ Yes [1]

□ No [0]

☐ DON’T KNOW [888]

☐ REFUSE [9]

[If 2 = NO/DK/REFUSE, skip to question 6]

|  | **Yes** | **No** | **DON’T KNOW** | **REFUSE** |
| --- | --- | --- | --- | --- |
| **3. Have you been thinking about how you might do this?** [c_cssr3] | ☐ [1] | ☐ [0] | ☐ [888] | ☐ [999] |
| **4. Have you had these thoughts and had some intention of acting on them?** [c_cssr4] | ☐ [1] | ☐ [0] | ☐ [888] | ☐ [999] |
| **5. Have you started to work out or worked out the details of how to kill yourself? Do you intend to carry out this plan?** [c_cssr5] | ☐ [1] | ☐ [0] | ☐ [888] | ☐ [999] |

**6. Have you ever done anything, started to do anything, or prepared to do anything to end your life?** [c_cssr6]

□ Yes [1]

□ No [0]

☐ DON’T KNOW [888]

☐ REFUSE [999]

[If 6 = NO/DK/REFUSE, skip to PTSD (PCL-5)]

**7. Was this within the past 3 months, from [DATEFILL] up to and including today?** [c_cssr7]

□ Yes [1]

□ No [0]

☐ DON’T KNOW [888]

☐ REFUSE [999]

[If (C-SSRS Question 4 = “YES” OR C-SSRS Question 5 = “YES” OR PHQ-9 Question 9 = “Nearly every day”) display this script for RA]

Participant responded “Yes” to items 4 OR 5 on C-SSRS OR responded “Nearly every day” to item 9 on the PHQ-9:

**RA Instructions:**

**Initiate 3-way call with suicide hotline, say:** “I’m concerned about you hurting yourself and I want to make sure you are safe and have the support you need. I am going to call a crisis hotline number for you. I will call you back at a later time to reschedule our appointment to continue your interview.”

**New Mexico Crisis and Access Line**
24 hours a day, 7 days a week, 365 days a year
1-855-NMCRISIS (662-7474); TTY 1-855-227-5485;
711 for relay (hearing & speech impaired)

**Didi Hirsch’s Suicide Prevention Crisis Line (LA Sites)**

24 hours a day, 7 days a week, 365 days a year
[988](tel:1-988) or 1-800-273-8255,

**National Suicide Prevention Lifeline**
1-800-273-TALK

**Stay on the phone with the participant until s/he receives help from Crisis Line**

**Notify one of the PIs below:**

Please remember you will need to fill out an Incident Report Form within 24 hours of this event.

[If (C-SSRS Question 4 = “No” AND C-SSRS Question 5 = “No”) AND (PHQ-9 Question 9 = “Several days” OR “More than half the days” OR “DON’T KNOW” OR “REFUSE”) display this script for RA]

Participant responds “No” to items 4 AND 5 on C-SSRS, BUT to item 9 on the PHQ-9 answers “Several days” OR “More than half the days” OR “Nearly every day” OR “DON’T KNOW” OR “REFUSE”:

**RA Instructions:**

Say: “You mentioned you’ve been having a tough time recently. I want to give you some information about how to get help for different kinds of problems. OFFER GUIDE AND CIRCLE RELEVANT RESOURCES. You can also talk with your medical provider or someone at x clinic about how you’ve been feeling.”

**If participant endorses more imminent danger:**

**Initiate 3-way call with suicide hotline, say:** "I'm concerned about you hurting yourself and I want to make sure you are safe and have the support you need. I am going to call a crisis hotline number for you. I will call you back at a later time to reschedule our appointment to continue your interview."

**New Mexico Crisis and Access Line**
24 hours a day, 7 days a week, 365 days a year
1-855-NMCRISIS (662-7474); TTY 1-855-227-5485;
711 for relay (hearing & speech impaired)

**Didi Hirsch’s Suicide Prevention Crisis Line (LA Sites)**

24 hours a day, 7 days a week, 365 days a year
[988](tel:1-988) or 1-800-273-8255,

**National Suicide Prevention Lifeline**
1-800-273-TALK

**Stay on the phone with the participant until s/he receives help from Crisis Line**

**Notify one of the PIs below:**

Please remember you will need to fill out an Incident Report Form within 24 hours of this event.

**Post-Traumatic Stress Disorder (PCL-5)**

**Sometimes things happen to people that are unusually or especially frightening, horrible, or traumatic. For example:**

- **a serious accident or fire**
- **a physical or sexual assault or abuse**
- **an earthquake or flood**
- **a war**
- **seeing someone be killed or seriously injured**
- **having a loved one die through homicide or suicide.**

**1. Have you ever experienced this kind of event?** [c_pcl1]

☐ Yes [1]

☐ No [0]

☐ DON’T KNOW [888]

☐ REFUSE [999]

[If 1 = NO/DK/REFUSE, skip to Experience with Care Coordinator)]

**I’m going to read a list of problems that people sometimes have in response to a very stressful experience. Please answer a few questions about your worst event, which means the event that currently bothers you the most. This could be one of the examples mentioned before or some other very stressful experience. Also, it could be a single event (for example, a car crash) or multiple similar events (for example, multiple stressful events in a war-zone or repeated sexual abuse).**

**2. Briefly identify the worst event; the one that currently bothers you the most. I don't need a lot of detail or the full story, but I do need to know what type of experience it was. If you are assigned a care coordinator for this study, I will share a short description of your worst event with them so that you don’t have to repeat the conversation with them; I won’t share any other details about your answers to these questions.**

**_______________________________________**

[Record a simple description of the worst event here, in the patient’s own words – e.g., “Car accident three years ago” or “Sexual assault by a family member growing up.” If the patient shares intimate details about the event, do NOT record them.]

☐ DON’T KNOW [888]

☐ REFUSE [999]

**Keeping your worst event in mind, please let me know how much you have been bothered by each problem in the past 30 days. In the past 30 days, from [DATEFILL] up to and including today, how much were you bothered by:**

|  | **Not at all** | **A little bit** | **Moder-ately** | **Quite a bit** | **Extreme-ly** | **DON’T KNOW** | **REFUSE** |
| --- | --- | --- | --- | --- | --- | --- | --- |
| **3. Repeated, disturbing, and unwanted memories of the stressful experience?** [c_pcl3] | ☐[0] | ☐ [1] | ☐[2] | ☐ [3] | ☐[4] | ☐ [888] | ☐[999] |
| **4. Repeated, disturbing dreams of the stressful experience?** [c_pcl4] | ☐ [0] | ☐ [1] | ☐ [2] | ☐ [3] | ☐ [4] | ☐ [888] | ☐ [999] |
| **5. Suddenly feeling or acting as if the stressful experience were actually happening again (as if you were actually back there reliving it)?** [c_pcl5] | ☐ [0] | ☐ [1] | ☐ [2] | ☐ [3] | ☐ [4] | ☐ [888] | ☐ [999] |
| **6. Feeling very upset when something reminded you of the stressful experience?** [c_pcl6] | ☐ [0] | ☐ [1] | ☐ [2] | ☐ [3] | ☐ [4] | ☐ [888] | ☐ [999] |
| **7. Having strong physical reactions when something reminded you of the stressful experience (for example, heart pounding, trouble breathing, sweating)?** [c_pcl7] | ☐ [0] | ☐ [1] | ☐ [2] | ☐ [3] | ☐ [4] | ☐ [888] | ☐ [999] |
| **8. Avoiding memories, thoughts, or feelings related to the stressful experience?** [c_pcl8] | ☐ [0] | ☐ [1] | ☐ [2] | ☐ [3] | ☐ [4] | ☐ [888] | ☐ [999] |
| **9. Avoiding external reminders of the stressful experience (for example, people, places, conversations, activities, objects, or situations)?** [c_pcl9] | ☐ [0] | ☐ [1] | ☐ [2] | ☐ [3] | ☐ [4] | ☐ [888] | ☐ [999] |
| **10. Trouble remembering important parts of the stressful experience?** [c_pcl10] | ☐[0] | ☐ [1] | ☐ [2] | ☐ [3] | ☐ [4] | ☐ [888] | ☐ [999] |
| **11. Having strong negative beliefs about yourself, other people, or the world (for example, having thoughts such as: I am bad, there is something seriously wrong with me, no one can be trusted, the world is completely dangerous)?** [c_pcl11] | ☐ [0] | ☐ [1] | ☐ [2] | ☐ [3] | ☐ [4] | ☐ [888] | ☐ [999] |
| **12. Blaming yourself or someone else for the stressful experience or what happened after it?** [c_pcl12] | ☐ [0] | ☐ [1] | ☐ [2] | ☐ [3] | ☐ [4] | ☐ [888] | ☐ [999] |
| **13. Having strong negative feelings such as fear, horror, anger, guilt, or shame?** [c_pcl13] | ☐ [0] | ☐ [1] | ☐ [2] | ☐ [3] | ☐ [4] | ☐ [888] | ☐ [999] |
| **14. Loss of interest in activities that you used to enjoy?** [c_pcl14] | ☐ [0] | ☐ [1] | ☐ [2] | ☐ [3] | ☐ [4] | ☐ [888] | ☐ [999] |
| **15. Feeling distant or cut off from other people?** [c_pcl15] | ☐ [0] | ☐ [1] | ☐ [2] | ☐ [3] | ☐ [4] | ☐ [888] | ☐ [999] |
| **16. Trouble experiencing positive feelings (for example, being unable to feel happiness or have loving feelings for people close to you)?** [c_pcl16] | ☐ [0] | ☐ [1] | ☐ [2] | ☐ [3] | ☐ [4] | ☐ [888] | ☐ [999] |
| **17. Irritable behavior, angry outbursts, or acting aggressively?** [c_pcl17] | ☐ [0] | ☐ [1] | ☐ [2] | ☐ [3] | ☐ [4] | ☐ [888] | ☐ [999] |
| **18. Taking too many risks or doing things that could cause you harm?** [c_pcl18] | ☐ [0] | ☐ [1] | ☐ [2] | ☐ [3] | ☐ [4] | ☐ [888] | ☐ [999] |
| **19. Being “superalert” or watchful or on guard?** [c_pcl19] | ☐ [0] | ☐ [1] | ☐ [2] | ☐ [3] | ☐ [4] | ☐ [888] | ☐ [999] |
| **20. Feeling jumpy or easily startled?** [c_pcl20] | ☐ [0] | ☐ [1] | ☐ [2] | ☐ [3] | ☐ [4] | ☐ [888] | ☐ [999] |
| **21. Having difficulty concentrating?** [c_pcl21] | ☐ [0] | ☐ [1] | ☐ [2] | ☐ [3] | ☐ [4] | ☐ [888] | ☐ [999] |
| **22. Trouble falling or staying asleep?** [c_pcl22] | ☐ [0] | ☐ [1] | ☐ [2] | ☐[3] | ☐ [4] | ☐ [888] | ☐ [999] |

**The next questions ask about treatment and counseling that you have received. All of your answers are confidential, and we will not share this information with anyone outside of the research project.**

**Experience with Care Coordinator**

**1. In the past 12 months, from [DATEFILL] up to and including today, have you worked with a care coordinator? A care coordinator is a person who helps you move through the steps of treatment, keeps track of your progress, and works closely with your medical team to help you achieve your goals on your plan of care. They might also help with resources such as food or transportation, to name a few. They are sometimes also called a caseworker, care navigator, peer support worker, care manager, case manager, community support worker, or community health worker. The care coordinator might work for [CLINIC NAME], a health insurance company, or some other health care organization.** [c_care1]

☐ Yes [1]

☐ No [0]

☐ DON’T KNOW [888]

☐ REFUSE[999]

[If 1 = NO/DK/REFUSE, skip to Mental Health Treatment]

**2. In the past 12 months, how many care coordinators have you worked with?** [c_care2]

_____ days [RANGE: 0 - 9]

☐ DON’T KNOW [888]

☐ REFUSE [999]

**3. In the past 12 months, what kind of problems did your care coordinator(s) help you with?** [c_care3] [derived variable = c_care3dv]

**[DO NOT READ THE RESPONSE OPTIONS TO THE PARTICIPANT]**

**[CHECK ALL THAT APPLY]**

☐ PHYSICAL HEALTH/MEDICAL [c_care3___1] [1]

☐ MENTAL HEALTH [c_care3___2] [2]

☐ SUBSTANCE USE[c_care3___3] [3]

☐ HOUSING OR HOMELESSNESS[c_care3___4][4]☐ TRANSPORTATION[c_care3___5][5]

☐ CHILD CARE OR OTHER CAREGIVING RESPONSIBILITIES[c_care3___6] [6]

☐ PAYING FOR FOOD, BILLS, ETC. [c_care3___7] [7]

☐ EMPLOYMENT[c_care3___8] [8]

☐ EDUCATION[c_care3___9] [9]☐ SAFETY/DOMESTIC VIOLENCE[c_care3___10] [10]

☐ LEGAL OR IMMIGRATION ISSUES[c_care3___11] [11]☐ DON’T KNOW[c_care3___888][888]

☐ REFUSE[c_care3___999] [999]

**4. In the past 12 months, in what ways did you talk with your care coordinator(s)?** **[CHECK ALL THAT APPLY]** **[**c_care4] [derived variable = c_care4_dv]

☐ In-person, at a health clinic or organization [c_care4___1] [1]

☐ In-person, at home, or in the community [c_care4___2] [2]

☐ Telephone [c_care4___3] [3]

☐ E-mail or text message [c_care4___4] [4]

☐ DON’T KNOW [c_care4___888] [888]

☐ REFUSE [c_care4___999] [999]

**5. In the past 12 months, how often did you talk with your care coordinator(s)? (If you have more than one care coordinator, please provide an overall answer for how often you talk with any of your care coordinators).** [c_care5]

☐ Once a week or more [1]

☐ 1-3 times a month [2]

☐ Less than once a month [3]

☐ DON’T KNOW [888]

☐ REFUSE [999]

**Mental Health Treatment**

**1a. In the past 30 days, from [DATEFILL] up to and including today, have you received professional therapy or counseling for depression or difficulties with your mood?** [c_mht1]

☐ Yes [1]

☐ No [0]

☐ DON’T KNOW [888]

☐ REFUSE [999]

**1b. In the past 30 days, from [DATEFILL] up to and including today, have you received professional therapy or counseling for PTSD (Post-Traumatic Stress Disorder) or difficulties related to a traumatic event?** [c_mht2]

☐ Yes [1]

☐ No [0]

☐ DON’T KNOW [888]

☐ REFUSE [999]

**2a. In the past 30 days, have you taken prescription medication that was prescribed for depression or difficulties with your mood?** [c_mht3]

☐ Yes [1]

☐ No [0]

☐ DON’T KNOW [888]

☐ REFUSE [999]

**2b. In the past 30 days, have you taken prescription medication that was prescribed for PTSD or difficulties related to a traumatic event?** [c_mht4]

☐ Yes [1]

☐ No [0]

☐ DON’T KNOW [888]

☐ REFUSE [999]

**History of MOUD**

**1. Have you ever received any treatment for problems with using heroin or pain pills? For example, therapy, counseling, or medication?** [c_moud1]

☐ No [1]

☐ Yes, counseling only [2]

☐ Yes, medication only [3]

☐ Yes, counseling and medication [4]

☐ DON’T KNOW [888]

☐ REFUSE [999]

**2. Have you ever taken Buprenorphine (Suboxone, Zubsolv, Subutex, Sublocade) to treat your problems with using heroin, fentanyl, or pain pills?** [c_moud2]

**☐ Yes [No codebook response listed for this question]**

**☐ No [No codebook response listed for this question]**

**[If participant does not endorse use of Buprenorphine (Suboxone, Zubsolv, Subutex, Sublocade), please enter "No" for Question 2]**

[If 1 = NO/COUNSELING ONLY/DK/REFUSE, skip to Support]

**3. Have you ever taken any other medications to treat your problems with using heroin/fentanyl or pain pills?**

|  | **Yes** | **No** | **DON’T KNOW** | **REFUSE** |
| --- | --- | --- | --- | --- |
| **3a. Methadone**[c_moud3a] | ☐ [1] | ☐ [0] | ☐ [888] | ☐ [999] |
| **3b. Injectable Naltrexone (Vivitrol)** [c_moud3b] | ☐ [1] | ☐ [0] | ☐ [888] | ☐ [999] |
| **3c. Other**[c_moud3c] | ☐ [1] | ☐ [0] | ☐ [888] | ☐ [999] |
| **IF OTHER, which medication(s)?** [c_moud3_other]**__________________** | | | | |

[If 2 and 3a = NO/DK/REFUSE, skip to Question 5]

**4. When you took these medications, were they ever prescribed for you?**

**(If participant needs clarification, say “sometimes people get these medicines from a prescription and sometimes they get them on the street. I’m asking if you have gotten any of these from a prescription.”)**

|  | **No, never** | **Some-times, but not always** | **Yes, always** | **DON’T KNOW** | **REFUSE** |
| --- | --- | --- | --- | --- | --- |
| **4a. Buprenorphine (Suboxone, Zubsolv, Subutex, Sublocade)** [c_moud4a] | ☐ [0] | ☐ [1] | ☐ [2] | ☐ [888] | ☐ [999] |
| **4b. Methadone**[c_moud4b] | ☐ [0] | ☐ [1] | ☐ [2] | ☐ [888] | ☐ [999] |

[If 2 = YES, display Question 4a]

[If 3a = YES, display Question 4b]

**5. Which medication have you taken in the past 30 days, from [DATEFILL] up to and including today, to treat your use of heroin/fentanyl or prescription pain pills?**

|  | **Yes** | **No** | **DON’T KNOW** | **REFUSE** |
| --- | --- | --- | --- | --- |
| **5a. Buprenorphine (Suboxone, Zubsolv, Subutex, Sublocade)** [c_moud5a] | ☐ [1] | ☐ [0] | ☐ [888] | ☐ [999] |
| **5b. Methadone**[c_moud5b] | ☐ [1] | ☐ [0] | ☐ [888] | ☐ [999] |
| **5c. Injectable Naltrexone (Vivitrol)** [c_moud5c] | ☐ [1] | ☐ [0] | ☐ [888] | ☐ [999] |
| **5d. Other**[c_moud5d] | ☐ [1] | ☐ [0] | ☐ [888] | ☐ [999] |
| **IF OTHER, which medication(s)?** [c_moud5_other] **__________________** | | | | |

[If 5a or 5b = NO/DK/REFUSE, skip to Support]

**6. When you took these medications in the past 30 days, were they prescribed for you?**

**(If participant needs clarification, say “sometimes people get these medicines from a prescription and sometimes they get them on the street. I’m asking if you have gotten any of these from a prescription.”)**

|  | **No, never** | **Some-times, but not always** | **Yes, always** | **DON’T KNOW** | **REFUSE** |
| --- | --- | --- | --- | --- | --- |
| **6a. Buprenorphine (Suboxone, Zubsolv, Subutex, Sublocade)** [c_moud6a] | ☐ [0] | ☐ [1] | ☐ [2] | ☐ [888] | ☐ [999] |
| **6b. Methadone**[c_moud6b] | ☐ [0] | ☐ [1] | ☐ [2] | ☐ [888] | ☐ [999] |

[If 5a = YES, display Question 6a]

[If 5b = YES, display Question 6b]

[If 5a = NO/DK/REFUSE, skip to Support]

**7. Have you been taking buprenorphine (Suboxone, Zubsolv, Subutex, Sublocade, etc.) for at least the past 3 months, from [DATEFILL] up to and including today, to treat your use of heroin/fentanyl or prescription pain pills?** [c_moud7]

☐ Yes [1]

☐ No [0]

☐ DON’T KNOW [888]

☐ REFUSE [999]

[If 7 = NO/DK/REFUSE, skip to Support]

**8. In the past 3 months while you were taking buprenorphine (Suboxone, Zubsolv, Subutex, Sublocade, etc.), did you ever stop taking the medication for more than 7 days in a row?** [c_moud8]

☐ Yes [1]

☐ No [0]

☐ DON’T KNOW [888]

☐ REFUSE [999]

**9. When you get your prescription for buprenorphine (Suboxone, Zubsolv, Subutex, Sublocade, etc.), do you usually get the prescription from [HEALTH SYSTEM]? (If participant needs clarification, say “Many patients who take buprenorphine have a doctor or clinic that they regularly get the prescription from. I am asking if [HEALTH SYSTEM] is where you get your prescriptions.”)** [c_moud9]

☐ Yes, I regularly get my buprenorphine prescription from [HEALTH SYSTEM [1]

☐ No, I regularly get my buprenorphine prescription from some other clinic or health system[0]

☐ Not applicable, I do not have a regular provider for buprenorphine [2]

☐ DON’T KNOW [888]

☐ REFUSE [999]

**These last questions ask about what is going on in your life outside of treatment or counseling. All of your answers are confidential, and we will not share this information with anyone outside of the research project.**

**Questions about support persons**

**1. Do you have an adult person in your life who you regularly connect with and who is supportive of you? This is someone that you see, call, text or otherwise connect with at least three times a week.** [c_support1]

☐ Yes [1]

☐ No [0]

☐ DON’T KNOW [888]

☐ REFUSE [999]

[If 1 = NO/DK/REFUSE, skip to Homelessness]

**2. Does your main support person currently use heroin or have a problem with pain pills or other opioids?** [c_support2]

☐ Yes [1]

☐ No [0]

☐ DON’T KNOW [888]

☐ REFUSE [999]

**Homelessness (Homelessness Screening Clinical Reminder Tool/SAMHSA GPRA)**

**1. In the past 3 months, from [DATEFILL] up to and including today, have you been living in stable housing that you own, rent, or stay in as part of a household?** [c_home1]

☐ Yes [1]

☐ No [0]

☐ DON’T KNOW [888]

☐ REFUSE [999]

[If 1 = YES, skip to question 3]

**2. In the past 3 months, where have you been living most of the time?**

**[DO NOT READ RESPONSE OPTIONS TO PARTICIPANT]** [c_home2]

☐ SHELTER (SAFE HAVENS, TRANSITIONAL LIVING CENTER, LOW-DEMAND [1] FACILITIES, RECEPTION CENTERS, OTHER TEMPORARY DAY OR EVENING FACILITY)

☐ STREET/OUTDOORS (SIDEWALK, DOORWAY, PARK, PUBLIC OR ABANDONED BUILDING) [2]

☐ INSTITUTION (HOSPITAL, NURSING HOME, JAIL/PRISON) [3]

☐ OWN/RENT APARTMENT, ROOM OR HOUSE [4]

☐ SOMEONE ELSE’S APARTMENT, ROOM OR HOUSE [5]

☐ DORMITORY/COLLEGE RESIDENCE [6]

☐ HALFWAY HOUSE [7]

☐ RESIDENTIAL TREATMENT FACILITY/PROGRAM [8]

☐ OTHER [9]

☐ DON’T KNOW [888]

☐ REFUSE [999]

**IF OTHER, specify:** [c_home2_other]**__________________**

**3. Are you worried or concerned that in the next 3 months, you may (still) not have stable housing that you own, rent, or stay in as part of a household?** [c_home3]

☐ Yes [1]

☐ No [0]

☐ DON’T KNOW [888]

☐ REFUSE [999]

**Legal Involvement (National Survey on Drug Use and Health)**

**The next questions are about encounters with the police or the court system.**

**1. Not counting minor traffic violations, have you ever been arrested and booked for breaking the law? Being “booked” means that you were taken into custody and processed by the police or someone connected with the courts, even if you were then released.** [c_legal1]

☐ Yes [1]

☐ No [0]

☐ DON’T KNOW [888]

☐ REFUSE [999]

[If 1 = NO/DK/REFUSE, skip to Patient Reported Experience]

**2. Not counting minor traffic violations, how many times during the past 12 months, from [DATEFILL] up to and including today, have you been arrested and booked for breaking a law?** [c_legal2]

_____ times [RANGE: 0 – 99]

☐ DON’T KNOW [888]

☐ REFUSE [999]

**3. Were you on probation, parole, supervised release, or other conditional release from prison at any time during the past 12 months?** [c_legal3]

☐ Yes [1]

☐ No [0]

☐ DON’T KNOW [888]

☐ REFUSE [999]

**4. Are you presently awaiting charges, trial, or sentence?** [c_legal4]

☐ Yes [1]

☐ No [2]

☐ DON’T KNOW [888]

☐ REFUSE [999]

**Patient-reported experience (VR-12)**

**This questionnaire asks for your views about your health. This information will help keep track of how you feel and how well you are able to do your usual activities.**

**1. In general, would you say your health is:** [c_vr1]

☐ Excellent [1]

☐ Very good [2]

☐ Good [3]

☐ Fair [4]

☐ Poor [5]

☐ DON’T KNOW [888]

☐ REFUSE [999]

**The following questions are about activities you might do during a typical day. Does your health now limit you in these activities? If so, how much?**

**2a. Moderate activities, such as moving a table, pushing a vacuum cleaner, bowling, or playing golf?** [c_vr2a]

☐ Yes, limited a lot [1]

☐ Yes, limited a little [2]

☐ No, not limited at all [3]

☐ DON’T KNOW [888]

☐ REFUSE [999]

**2b. Climbing several flights of stairs?** [c_vr2b]☐ Yes, limited a lot [1]

☐ Yes, limited a little [2]

☐ No, not limited at all [3]

☐ DON’T KNOW [888]

☐ REFUSE [999]

**During the past 30 days, from [DATEFILL] up to and including today, have you had any of the following problems with your work or other regular daily activities as a result of your physical health?**

**3a. Accomplished less than you would like.** [c_vr3a]

☐ No, none of the time [1]

☐ Yes, a little of the time [2]

☐ Yes, some of the time [3]

☐ Yes, most of the time [4]

☐ Yes, all of the time [5]

☐ DON’T KNOW [888]

☐ REFUSE [999]

**3b. Were limited in the kind of work or other activities.** [c_vr3b]

☐ No, none of the time [1]

☐ Yes, a little of the time [2]

☐ Yes, some of the time [3]

☐ Yes, most of the time [4]

☐ Yes, all of the time [5]

☐ DON’T KNOW [888]

☐ REFUSE [999]

**During the past 30 days, from [DATEFILL] up to and including today, have you had any of the following problems with your work or other regular daily activities as a result of any emotional problems (such as feeling depressed or anxious)?**

**4a. Accomplished less than you would like.** [c_vr4a]

☐ No, none of the time [1]

☐ Yes, a little of the time [2]

☐ Yes, some of the time [3]

☐ Yes, most of the time [4]

☐ Yes, all of the time [5]

☐ DON’T KNOW [888]

☐ REFUSE [999]

**4b. Didn't do work or other activities as carefully as usual.** [c_vr4b]

☐ No, none of the time [1]

☐ Yes, a little of the time [2]

☐ Yes, some of the time [3]

☐ Yes, most of the time [4]

☐ Yes, all of the time [5]

☐ DON’T KNOW [888]

☐ REFUSE [999]

**5. During the past 30 days, how much did pain interfere with your normal work (including both work outside the home and house work)?** [c_vr5]

☐ Not at all [1]

☐ A little bit [2]

☐ Moderately [3]

☐ Quite a bit [4]

☐ Extremely [5]

☐ DON’T KNOW [888]

☐ REFUSE [999]

**These questions are about how you feel and how things have been with you during the past 30 days, from [DATEFILL] up to and including today. How much of the time during the past 30 days:**

|  | **All of the time** | **Most of the time** | **A good bit of the time** | **Some of the time** | **A little bit of the time** | **None of the time** | **DON’T KNOW** | **REFUSE** |
| --- | --- | --- | --- | --- | --- | --- | --- | --- |
| **6a. Have you felt calm and peaceful?** [c_vr6a] | ☐ [1] | ☐ [2] | ☐[3] | ☐ [4] | ☐[5] | ☐ [6] | ☐ [888] | ☐[999] |
| **6b. Did you have a lot of energy?** [c_vr6b] | ☐ [1] | ☐ [2] | ☐ [3] | ☐ [4] | ☐ [5] | ☐ [6] | ☐ [888] | ☐ [999] |
| **6c. Have you felt downhearted and blue?** [c_vr6c] | ☐ [1] | ☐[2] | ☐ [3] | ☐ [4] | ☐ [5] | ☐ [6] | ☐[888] | ☐ [999] |

**7. During the past 30 days, from [DATEFILL] up to and including today, how much of the time has your physical health or emotional problems interfered with your social activities (like visiting with friends, relatives, etc.)?** [c_vr7]

☐ All of the time [1]

☐ Most of the time [2]

☐ Some of the time [3]

☐ A little of the time [4]

☐ None of the time [5]

☐ DON’T KNOW [888]

☐ REFUSE [999]

**Now, I'd like to ask you some questions about how your health may have changed.**

**8. Compared to one year ago, from [DATEFILL] up to and including today, how would you rate your physical health in general now?** [c_vr8]

☐ Much better [1]

☐ Slightly better [2]

☐ About the same [3]

☐ Slightly worse [4]

☐ Much worse [5]

☐ DON’T KNOW[888]

☐ REFUSE [999]

**9. Compared to one year ago, from [DATEFILL] up to and including today, how would you rate your emotional problems (such as feeling anxious, depressed or irritable) now?** [c_vr9]

☐ Much better [1]

☐ Slightly better [2]

☐ About the same [3]

☐ Slightly worse [4]

☐ Much worse [5]

☐ DON’T KNOW [888]

☐ REFUSE [999]

**Disability and impairment (SDS)**

**In the past week, from [DATEFILL] up to and including today, how much have your drug/alcohol use and mental health symptoms disrupted:**

|  | **Not at all** | **Mildly** | | | **Moderately** | | | **Markedly** | | | **Extremely** |  | |
| --- | --- | --- | --- | --- | --- | --- | --- | --- | --- | --- | --- | --- | --- |
|  | **0** | **1** | **2** | **3** | **4** | **5** | **6** | **7** | **8** | **9** | **10** | **DON’T KNOW** | **REFUSE** |
| **1. Your work/ schoolwork? (Work includes paid, unpaid volunteer work or training)**  [c_sds1] | ☐  [0] | ☐  [1] | ☐  [2] | ☐  [3] | ☐  [4] | ☐  [5] | ☐  [6] | ☐  [7] | ☐  [8] | ☐  [9] | ☐  [10] | ☐ [888] | ☐[999] |
| **2. Your social life/leisure activities?**  [c_sds2] | ☐  [0] | ☐  [1] | ☐  [2] | ☐  [3] | ☐  [4] | ☐  [5] | ☐  [6] | ☐  [7] | ☐  [8] | ☐  [9] | ☐  [10] | ☐ [888] | ☐ [999] |
| **3. Your family life/home responsibilities?** [c_sds3] | ☐  [0] | ☐  [1] | ☐  [2] | ☐  [3] | ☐  [4] | ☐  [5] | ☐  [6] | ☐  [7] | ☐  [8] | ☐  [9] | ☐  [10] | ☐ [888] | ☐ [999] |

**To wrap up, we need to collect a few personal details from you. This information** **will be kept separate from your responses to all the other questions.**

**(If participants have questions, can explain: This information is used to create an anonymous, unique identifying participant record number for your data, as required by the National Institutes of Health. We will also use it to gather data from public records.)**

**What was your full legal name at birth?**

First: _________________ Middle: _________________ Last: _________________

☐ DON’T KNOW

☐ REFUSE

**Do you have the same legal name now?**

☐ Yes

☐ No

☐ DON’T KNOW

☐ REFUSE

[If “Do you have the same legal name” = NO/DK/REFUSE, ask for current full legal name]

**What is your full legal name now?**

First: _________________ Middle: _________________ Last: _________________

☐ DON’T KNOW

☐ REFUSE

**What is your father’s last name? This should be the father listed on your birth certificate.**

Father’s surname: ____________________

☐ DON’T KNOW

☐ REFUSE

**What state or country were you born in?**

_________ [U.S. state, DC, PR, Virgin Islands, Guam, Canada, Cuba, Mexico, or rest of world]

☐ DON’T KNOW

☐ REFUSE

**What city or municipality were you born in?**

_________

☐ DON’T KNOW

☐ REFUSE

**END OF INTERVIEW**
